# Dynamic survival analysis for non-Markovian epidemic models

**DOI:** 10.1098/rsif.2022.0124

**Published:** 2022-06-01

**Authors:** Francesco Di Lauro, Wasiur R. KhudaBukhsh, István Z. Kiss, Eben Kenah, Max Jensen, Grzegorz A. Rempała

**Affiliations:** ^1^ Big Data Institute, University of Oxford, Oxford, OX3 7LF, UK; ^2^ Department of Mathematics, University of Nottingham, Nottingham, NG7 2RD, UK; ^3^ Department of Mathematics, University of Sussex, Brighton, BN1 9RH, UK; ^4^ Department of Biostatistics, The Ohio State University, Columbus, OH 43210, USA

**Keywords:** spatial epidemic models, parameter inference, MCMC methods, survival analysis

## Abstract

We present a new method for analysing stochastic epidemic models under minimal assumptions. The method, dubbed dynamic survival analysis (DSA), is based on a simple yet powerful observation, namely that population-level mean-field trajectories described by a system of partial differential equations may also approximate individual-level times of infection and recovery. This idea gives rise to a certain non-Markovian agent-based model and provides an agent-level likelihood function for a random sample of infection and/or recovery times. Extensive numerical analyses on both synthetic and real epidemic data from foot-and-mouth disease in the UK (2001) and COVID-19 in India (2020) show good accuracy and confirm the method’s versatility in likelihood-based parameter estimation. The accompanying software package gives prospective users a practical tool for modelling, analysing and interpreting epidemic data with the help of the DSA approach.

## 1. Introduction

The standard approach to building a stochastic compartmental epidemic model is to make use of a continuous-time Markov chain (CTMC) to keep track of the sizes of the compartments over time (e.g. number of individuals with different immunological statuses) using counting processes [1]. Following the random time change representation of Poisson processes [2,3], the trajectory equations for those counting processes are written in terms of independent, unit rate Poisson processes. When the size of the population under consideration is large, those counting processes, appropriately scaled, converge to deterministic, continuous real-valued functions satisfying certain ordinary differential equations (ODEs) by virtue of the functional law of large numbers (FLLN) for Poisson processes [4,5]. This convergence provides a link between the stochastic and the deterministic world and the limiting ODEs are often referred to as the mean-field equations in the literature. Famous examples include the classical Kermack–McKendrick equations for the susceptible–infected–recovered (SIR) epidemic model [6].

However, this astounding popularity of the standard Markov models or the corresponding mean-field ODE models seems to belie their apparent lack of faithfulness to the underlying biology of the disease. To quote van Kampen [7],

‘Non-Markov is the rule, Markov is the exception’.

**Figure 1.**
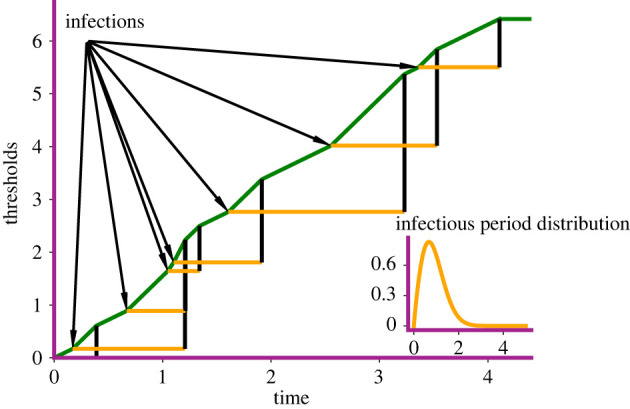
Sellke construction. Here, we begin with a single infected individual. The arrows point to the times of infection. The orange horizontal lines indicate the infectious period of each infected individual. The probability density function (PDF) of the infectious periods is shown in the inset (Weibull with shape *c* = 1.9 and scale 1).

Indeed, the population count-based Markov models assume exponentially distributed inter-event times. As a consequence, the instantaneous rates of infection and recovery are assumed constant regardless of key epidemiologically relevant covariates, such as the age of infection (see §2), time since vaccination, etc. As shown in [8] (in particular, see table 1 and fig. 1), the estimates obtained by assuming a Markovian model when the underlying model is non-Markovian could be significantly biased. While there are more advanced stochastic models that do incorporate those covariates (as we will also do in this paper), those models are often fit to data in an ad hoc fashion; or are too computationally expensive to be useful for practical purposes. Our aim in this work is to build a principled and rigorous statistical approach to fitting those more advanced stochastic models to data without compromising on simplicity.

In this paper, we present a survival analytic approach, dubbed dynamic survival analysis (DSA), that constructs probability distributions of individual times of infection and recovery from population-level (mean-field) trajectory equations. In [9], a subset of the authors first employed this idea in the context of the classical Kermack–McKendrick Markovian (SIR) epidemics described by their mean-field ODEs. Here, we extend the idea to the vastly more realistic class of non-Markovian models that allow non-exponential contact interval [8] and infectious periods. The theoretical underpinning is laid down by an extension of the so-called *Sellke construction* [1,10], which we describe in detail in §2.2.

There are several advantages of DSA. First, DSA does not require knowledge of the size of the susceptible population, which is almost always unknown in real epidemics and often assumed to be the population of the entire city, state, or even a country. In fact, DSA not only avoids this ad hoc adjustment, but also provides a ready estimate of the *effective population size*, tracking of which could provide further insights into an ongoing epidemic. Second, DSA does not require the whole epidemic trajectory and works with only a random sample of infection and, if available, recovery times. Third, on the strength of its survival analytic foundation, DSA is able to handle censoring, truncation and aggregation of data (over time and population) in a straightforward manner. We illustrate some of these features of the DSA method below.

The rest of the paper is structured as follows. §2 describes the stochastic model in terms of measure-valued processes and the so-called Sellke construction, along with their large population mean-field approximations. In §3, we describe the DSA modelling approach in detail before conducting extensive numerical analysis in §4. We apply the DSA method to the epidemics of foot-and-mouth disease (FMD) in the UK and COVID-19 in India. In §4, we also provide synthetic data analysis so that DSA could be compared against the true data-generating model. Finally, we conclude with a short discussion in §5. For the sake of completeness, additional mathematical derivations and numerical figures are provided in appendices, where we also compare the performances of Markovian and non-Markovian DSA on the FMD dataset.

## 2. Stochastic model

Because we want to keep track of important epidemiological covariates along with counts of individuals in different compartments, our primary tool will be measure-valued processes, which are naturally capable of carrying more information than raw population counts. The measure-valued representation will also allow us to turn an inherently non-Markovian model into a Markov model, albeit on a more abstract state space. While the age of infection (§2) is the most natural choice for ‘age’, one may also use the notion of age to account for other important covariates that describe time since some specific event. For instance, the biological age, time since vaccination are important for certain infectious diseases. Therefore, we use the term ‘age’ in a broad sense and keep track of the ages of individuals with different immunological statuses (susceptible, infected, recovered/removed).

Suppose we have *n* susceptible and *m* infected individuals initially. We assume that *m* depends on *n* in the sense that *m*/*n* → *ρ* as *n* → ∞ for some *ρ* ∈ (0, 1). Let us now define the following stochastic processes:

2.1 $$X_{t}^{S}\;:=\sum_{k=1}^{N_{S}(t)}\delta_{s_{k(t)}},\;X_{t}^{I}\;:=\sum_{k=1}^{N_{I}(t)}\delta_{i_{k(t)}},\;X_{t}^{R}\;:=\sum_{k=1}^{N_{R}(t)}\delta_{r_{k(t)}},$$

where *N*_*S*_(*t*), *N*_*I*_(*t*) and *N*_*R*_(*t*) are, respectively, the total numbers of susceptible, infected and recovered individuals in the population at time *t*. The quantities *s*_*k*_(*t*), *i*_*k*_(*t*) and *r*_*k*_(*t*) are the ages of the *k*th susceptible, infected and recovered individuals (following some specific ordering convention). The set-function *δ*_*x*_ is the Dirac measure, i.e. for a set *A*, the function *δ*_*x*_(*A*) takes value 1 if *x* ∈ *A* and 0 otherwise. The stochastic processes $X_{t}^{S}$, $X_{t}^{I}$ and $X_{t}^{R}$ keep track of the age distribution of the population of individuals. For instance, taking the ‘age’ for the infected individuals to represent the age of infection, $X_{t}^{I}([3.5,7])$ gives us the number of infected individuals whose ages of infection lie in the set [3.5, 7]. Now, define the stochastic process

$$X_{t}\;:=(X_{t}^{S},X_{t}^{I},X_{t}^{R}).$$

The process *X*_*t*_ is a Markov process. Although we do not explicitly show the dependence of the stochastic process *X*_*t*_ on the initial size of the susceptible population *n*, it is worth keeping in mind.

### 2.1. Contact intervals and infectious periods

We adopt the pairwise model of [8] to describe the dynamics of the epidemic process under the stochastic mass-action set-up. There are two types of events: infection and natural recovery. In order to describe the intensities (of the Markov process *X*_*t*_) corresponding to these two types of events, let us introduce two functions: $\beta \;:\;R_{+}\times \;R_{+}\to R_{+}$ and $\gamma \;:\;R_{+}\to R_{+}$. The function *β*(*u*, *v*) describes the instantaneous intensity of an infectious contact between a susceptible individual of age *u* and an infectious individual of age *v*. That is, the probability that a susceptible individual of age *u* will be infected by an infectious individual of age *v* in the next *δt* time unit is *n*^−1^*β*(*u*, *v*)*δt* under the stochastic law of mass-action, where *δt* is assumed infinitesimally small. In the language of the pairwise model [8] of infectious diseases, the function *β* characterizes the probability law of the *contact intervals*. The function *γ* is the hazard function that characterizes the probability law of the *infectious period*. Note that neither of these two probability laws needs to be exponential, even though *X*_*t*_ itself is a Markov process (see [11] for a similar example in the context of a stochastic chemical reaction network (CRN)). The infection and natural recovery processes are assumed independent. We also assume that recovered individuals can no longer infect others or be infected.

The stochastic process *X*_*t*_ can be simulated by extending the standard Doob–Gillespie’s stochastic simulation algorithm (SSA) in a straightforward manner. An alternative approach to simulating individual trajectories is the Sellke construction, which also provides the theoretical underpinning to the DSA approach. For the sake of simplicity, we will assume in the following that the function *β*(*u*, *v*) depends only on the age *v* of the infected individual and not on the age *u* of the susceptible individual, i.e. *β*(*u*, *v*) = *β*(*v*). This will allow for a simpler and a more intuitive description of the Sellke construction. The general case of *β*(*u*, *v*) is considered in appendix A.

### 2.2. Sellke construction

The classical Sellke construction [1] provides an alternative individual-based description of the standard stochastic mass-action (SIR) epidemic model. It can be shown that the resultant epidemic process is equivalent to the original population-level stochastic model in the sense that the counts of individuals with different immunological statuses have the same probability law under both constructions. However, the crux of the Sellke construction is that it describes the epidemic process in terms of individual survival probabilities (i.e. for an initially susceptible individual, the probability of remaining susceptible until time *t*). This is useful for parameter inference. The classical Sellke construction can be adapted to the age-structured epidemic model of ours in a straightforward fashion.

To each of the initial *n* susceptible individuals, we assign a threshold, an exponentially distributed random variable with mean one. Let *U*_*i*_ denote the threshold corresponding to the *i*th susceptible individual. The random variables *U*_1_, *U*_2_, …, *U*_*n*_ are independent. Let *U*_(1)_, *U*_(2)_, …, *U*_(*n*)_ be the corresponding order statistics, i.e. *U*_(1)_ ≤ *U*_(2)_ ≤ … ≤ *U*_(*n*)_. Let us now define the *cumulative infection pressure*

2.2 $$A(t)\;:=\int_{0}^{t}\frac{1}{n}\sum_{k=1}^{N_{I}(u)}\beta (i_{k}(u))du=\int_{0}^{t}\langle X_{u}^{I},n^{-1}\beta \rangle du,$$

where, for a point measure $\nu =\sum_{i=1}^{n}\delta_{x_{i}}$ and a measurable function *f*, the notation 〈ν, *f*〉 denotes the integration of the function *f* with respect to the measure *ν*, i.e.

$$\langle \nu ,f\rangle \;:=\int f\;d\nu =\sum_{i=1}^{n}f\;(x_{i}).$$

The epidemic process proceeds as follows: The first infection occurs when the cumulative infection pressure exceeds the smallest individual threshold, i.e. when $A(t)\geq U_{\left(1\right)}$ for the first time; the second infection occurs when $A(t)\geq U_{\left(2\right)}$, and so on. Note that infected individuals recover following an infectious period that has a probability law characterized by the hazard function *γ*. Therefore, it is possible that the cumulative infection pressure becomes constant when the last infected individual recovers and there are no more infected individuals. Susceptible individuals whose thresholds are never exceeded by the cumulative infection pressure A(t) escape infection and never leave the susceptible compartment. Figure 1 provides a pictorial description of the Sellke construction.

Let us denote the time of infection of an initially susceptible individual by *T*_*I*_. In essence, the Sellke construction specifies an individual-level survival function: The probability that an initially susceptible individual *i* remains susceptible until time *t*, conditional on the history (filtration) $H_{t-}$ of the epidemic process, is given by

2.3 $$P(T_{I}>t|H_{t-})=P(U_{i}>A_{t}|H_{t-})=\exp(-A_{t})=\exp\left(-\int_{0}^{t}\langle X_{u}^{I},n^{-1}\beta \rangle du\right),$$

where $U_{i}∼\mathrm{Exponential}\;(1)$ is the threshold of the individual *i*. This survival probability will play a crucial role in devising the DSA-likelihood function. It is worth pointing out that the random variable *T*_*I*_ is improper because some individuals may escape infection with positive probability.

From the classical theory of stochastic epidemiology, we know that appropriately scaled population counts in CTMC-based epidemic models converge to solutions to ODEs in the large population (mean-field) limit [1]. They are a consequence of the FLLN-type approximation theorems for Markov processes [4,5]. The intuition is that the stochastic fluctuation, which is typically described in terms of a zero-mean martingale after a Doob–Meyer decomposition of the counting processes around the mean, vanishes in the limit. A similar intuition holds true for measure-valued Markov processes. Indeed, the scaled process *n*^−1^*X*_*t*_ converges to a vector of deterministic measure-valued functions in the limit of *n* → ∞. Furthermore, when the limiting measure-valued functions admit densities, it is possible to describe them using partial differential equation (PDE) (e.g. see [12,13] and appendices).

### 2.3. Mean-field limit

We are interested in the limit of the epidemic process as *n* → ∞ with *m*/*n* → *ρ*, for some *ρ* ∈ (0, 1). Therefore, in the limit, the total scaled population size is (1 + *ρ*). We scale the system this way because we wish to interpret the susceptible curve as a survival function, which takes the value of one at zero. We shall elaborate further on this point in §3 on DSA.

Under some technical assumptions on the intensities and the initial population size (more precise statement in appendix A), the scaled stochastic process *n*^−1^*X*_*t*_ converges to a deterministic continuous function $x_{t}\;:=(x_{t}^{S},x_{t}^{I},x_{t}^{R})$ as *n* → ∞, where the components $x_{t}^{S},x_{t}^{I}$ and $x_{t}^{R}$ are measure-valued functions. The main technical tools required to establish the convergence are borrowed from existing probability theory literature. In particular, similar techniques and derivations can be found in [12–16]. A brief, intuitive sketch of the proof of convergence of the scaled process *n*^−1^*X*_*t*_ to the deterministic function *x*_*t*_ is provided in appendix A for the sake of completeness.

The densities $y_{S}(t,∙),y_{I}(t,∙)$ and $y_{R}(t,∙)$ of $x_{t}^{S},x_{t}^{I}$ and $x_{t}^{R}$ satisfy the system of PDE given in (A 5) in appendix A. Because of our simplifying assumption *β*(*u*, *v*) = *β*(*v*), it makes sense to integrate out the age component for the susceptible and the recovered individuals. Therefore, by defining

$$z_{S}(t)\;:=\int_{0}^{\infty }y_{S}(t,s)ds,\mathrm{and}\;z_{R}(t)\;:=\int_{0}^{\infty }y_{R}(t,s)ds,$$

we can write the limiting system as follows:

2.4 $$\begin{array}{rl} & \frac{d}{dt}z_{S}(t)=-z_{S}(t)\int_{0}^{\infty }\beta (s)y_{I}(t,s)ds,\\ & \left(\partial_{t}+\partial_{s})y_{I}(t,s)=-\gamma (s)y_{I}(t,s\right)\\ \mathrm{and}\; & \frac{d}{dt}z_{R}(t)=\int_{0}^{\infty }\gamma (s)y_{I}(t,s)ds, \end{array}\}$$

with initial conditions *z*_*S*_(0) = 1, *z*_*R*_(0) = 0 and $y_{I}(0,∙)\;:\;R_{+}\to R_{+}$ such that

$$\int_{0}^{\infty }y_{I}(0,s)ds=\rho ,$$

and boundary condition

2.5 $$y_{I}(t,0)=z_{S}(t)\int_{0}^{\infty }\beta (s)y_{I}(t,s)ds.$$

Using the method of characteristics on (2.4), we get

$$y_{I}(t,s)=\left\{ \begin{array}{cc} \frac{y_{I}(0,s-t)S_{\gamma }(s)}{S_{\gamma }(s-t)}, & \;\mathrm{for}\;s>t,\\ y_{I}(t-s,0)S_{\gamma }(s), & \;\mathrm{for}\;t\geq s, \end{array}\right.$$

where $S_{\gamma }$ is the survival function of the probability distribution characterized by the hazard function *γ*. That is, $S_{\gamma }(t)=\exp\left(-\int_{0}^{t}\gamma (s)ds\right)$. Unfortunately, *y*_*I*_ does not admit an explicit solution. However, efficient numerical methods exist. We describe the solution scheme that we adopted in appendix B. The limiting proportion of recovered individuals *z*_*R*_ is also fully described by the limiting density *y*_*I*_ of infected individuals

$$z_{R}(t)=\int_{0}^{t}\int_{0}^{\infty }\gamma (v)y_{I}(u,v)dvdu=\int_{0}^{t}\langle x_{s}^{I},\gamma \rangle ds.$$

For different choices of the functions *β* and *γ* depending on the particular infectious disease in question, one can solve (2.4) numerically and fit to data. Typically, one would assume a parametric representation of the functions *β* and *γ,* and then attempt to infer those parameters based on data. However, a common problem in epidemiological literature is that the choice of the likelihood function is often ad hoc and strictly speaking, unjustifiable. To this end, the DSA method [9,17–20] provides, in a principled way, a likelihood function based on a random sample of transfer times.^1^ In the next section, we describe the DSA method in greater detail.

## 3. Dynamic survival analysis and parameter inference

The DSA method combines dynamical systems theory and survival analysis. For a given dynamical system, typically described by ODEs or PDEs for population counts/proportions, the DSA method provides an alternative interpretation that characterizes probability laws of transfer times [9,17,19,21]. The mathematical underpinning is provided by a novel application of the Sellke construction.

Rewriting (2.4) and with the initial condition *z*_*S*_(0) = 1, we immediately see

$$z_{S}(t)=\exp\left(-\int_{0}^{t}\int_{0}^{\infty }\beta (v)y_{I}(u,v)dvdu\right)=\exp\left(-\int_{0}^{t}\langle x_{s}^{I},\beta \rangle ds\right),$$

which is precisely the limit of the survival function $P(T_{I}>t|H_{t-})$ according to the Sellke construction in (2.3) as *n* → ∞. That is,

$$z_{S}(t)=\lim_{n\to \infty }P(T_{I}>t|H_{t-}).$$

Note that the random variable *T*_*I*_ in the Sellke construction depends on *n* even though we do not show it explicitly to keep the notations simple. Therefore, the function *z*_*S*_, the limiting proportion of susceptible individuals, can be interpreted as a survival function. However, the survival function *z*_*S*_ is improper because *z*_*S*_(∞) > 0. The quantity *z*_*S*_(∞) is precisely the limiting proportion of susceptible individuals that forever escape the infection. The survival function *z*_*S*_ can be made proper by conditioning on individuals who get infected [9]. Another important observation is that the ‘time to infection’ random variables associated with the initially susceptible individuals become independent in the limit of *n* → ∞. This phenomenon is sometimes referred to as *mean-field independence* [21,22].

### 3.1. Likelihood contribution of infection times

Let us denote by *θ* the set of parameters required to describe the contact interval distribution in terms of *β* and the infectious period in terms of *γ*. On account of the Sellke construction, we can treat the function *z*_*S*_ as an improper survival function for the (improper) random variable *T*_*I*_, the time to infection for an initially susceptible individual. Therefore, we can define the conditional probability density function (PDF)

3.1 $$f_{T,\theta }(t)\;:=-\frac{1}{\tau_{T}}\frac{d}{dt}z_{S}(t)=\frac{z_{S}(t)\langle x_{t}^{I},\beta \rangle }{\tau_{T}},$$

for the infection times, where *τ*_*T*_ : = 1 − *z*_*S*_(*T*). Also, set *τ* : = *τ*_∞_. The PDF *f*_*T*_ is proper by virtue of the conditioning.

Most epidemic and pandemic trajectories are only partially observed. A crucial advantage of the DSA approach is that it does not require the whole trajectory. Suppose we have a random sample of infection times *t*_1_, *t*_2_, …, *t*_*K*_ from an epidemic trajectory observed partially until time *T*, for some finite, positive number *T*. Then, following the mean-field independence, the contribution of the infection times to the DSA likelihood function is given by

3.2 $$\ell_{I}(\theta )\;:=\prod_{i=1}^{K}f_{T,\theta }(t_{i}).$$

The contribution ℓ_*I*_ can be modified in a straightforward fashion if the infection times are censored and/or truncated [19].

### 3.2. Likelihood contribution of recovery times

Now, let us describe the contribution of the recovery times to the DSA likelihood. While the recovery times are often not observed, or only partially observed (with further possibility of censoring or truncation), when available they can be incorporated into the DSA likelihood function, rendering it more informative. There are two possible scenarios. Let us consider the simpler case first: we have a random sample *s*_1_, *s*_2_, …, *s*_*L*_ of infectious periods. Then, denoting the PDF of the probability law characterized by the hazard function *γ* by *r*_*γ*_, the contribution of the random sample of infectious periods to the DSA likelihood function is given by

3.3 $$\ell_{R}^{\left(1\right)}(\theta )\;:=\prod_{i=1}^{L}r_{\gamma }(s_{i}).$$

Now, let us consider the second case: we do not directly observe individual infectious periods, but only observe recovery times. Suppose *u*_1_, *u*_2_, …, *u*_*M*_ is a random sample of recovery times of *M* individuals whose infection times are unknown. They are precisely a random sample of the sum of two independent random variables: time to infection and infectious period. Therefore, we can define the convolution-form PDF

3.4 $$g_{T,\theta }(t)\;:=\frac{g(t)}{\int_{0}^{T}g(s)ds},$$

conditional on the partially observed epidemic trajectory until time *T*, where

3.5 $$g(t)\;:=\int_{0}^{t}f_{T,\theta }(u)r_{\gamma }(t-u)du.$$

Now, with the conditional PDF of the recovery times given in (3.4), we can write down the contribution of the random sample *u*_1_, *u*_2_, …, *u*_*M*_ of recovery times as follows:

3.6 $$\ell_{R}^{\left(2\right)}(\theta )\;:=\prod_{i=1}^{M}g_{T,\theta }(u_{i}).$$

The conditional PDF *g*_*T*,*θ*_, in general, does not admit a closed-form expression. However, it can be computed numerically.

### 3.3. The DSA likelihood

Suppose we have a random sample *t*_1_, *t*_2_, …, *t*_*K*_ of infection times, a random sample *s*_1_, *s*_2_, …, *s*_*L*_ of infectious periods and a random sample *u*_1_, *u*_2_, …, *u*_*M*_ of recovery times. Then, the DSA likelihood function is given by

3.7 $$\ell (\theta )\;:=\ell_{I}(\theta )\times \ell_{R}^{\left(1\right)}(\theta )\times \ell_{R}^{\left(2\right)}(\theta ).$$

Note that it is not necessary to have data on recovery times. The likelihood contribution ℓ_*I*_(*θ*) is adequate for parameter inference. See [17], where parameter inference was done for the COVID-19 pandemic in the state of OH, USA, based only on infection times. When information on recovery times is unavailable, we simply set $\ell_{R}^{\left(1\right)}=1$ and $\ell_{R}^{\left(2\right)}=1$ by adopting the convention $\prod_{i=1}^{0}s_{i}=1$.

Often it is easier to work with the log-likelihood function. Therefore, for the purpose of parameter inference, we also define the DSA log-likelihood function

3.8 $$L(\theta )\;:=\log(\ell (\theta ))=\log(\ell_{I}(\theta ))+\log(\ell_{R}^{\left(1\right)}(\theta ))+\log(\ell_{R}^{\left(2\right)}(\theta )).$$

The maximum-likelihood estimate (MLE) $\hat{\theta }$ of the parameter *θ* is then numerically obtained by maximizing the log-likelihood function L(θ). That is,

3.9 $$\hat{\theta }\;:=\arg\max_{\theta }L(\theta ).$$

We present numerical results in §4. For Bayesian methods, we need to introduce a prior for the parameter *θ* and then implement a Markov Chain Monte Carlo (MCMC) algorithm to approximate the posterior distribution of the parameter *θ*. However, we do not pursue the Bayesian path in this paper.

### 3.4. Mean-field limits as Chapman–Kolmogorov equations

An alternative way to view DSA is to interpret the limiting trajectory equations as satisfying Chapman–Kolmogorov equations (written in the differential form) for certain probability distributions. Let us pick a random individual embedded in an infinitely large population (mean-field) and follow in time. Let $W(t)\in \{S,S^{c}\}$ denote a time-inhomogeneous CTMC that keeps track of whether an individual is in the susceptible compartment (S) or not ($S^{c}$). We specify the time-dependent instantaneous transition rates of *W*(*t*) as follows:

3.10 $$Q(t)\;:=\left(\begin{array}{cc} q_{SS}(t) & q_{SS^{c}}(t)\\ 0 & 0 \end{array}\right)=\left(\begin{array}{cc} -\langle x_{t}^{I},\beta \rangle & \left\langle x_{t}^{I},\beta \right\rangle \\ 0 & 0 \end{array}\right),$$

where *x*_*t*_ is the mean-field FLLN limit of the stochastic process *n*^−1^*X*_*t*_. Write $p_{t}\;:=(p_{t}^{S},p_{t}^{S^{c}})$ for $p_{t}^{A}\;:=P(W(t)=A)$, the marginal distribution of the Markov process *W*. We of course have $p_{t}^{S^{c}}=1-p_{t}^{S}$. Then, following the previous discussion, DSA, in essence, is tantamount to writing

$$p_{t}^{S}=\;\frac{z_{S}(t)}{1+\rho },\;p_{t}^{S^{c}}=\;\frac{1+\rho -z_{S}(t)}{1+\rho }.$$

It is straightforward to verify that *p*_*t*_ satisfies

3.11 $$\frac{d}{dt}\;p_{t}=p_{t}Q(t),$$

which is the time-inhomogeneous Chapman–Kolmogorov equation (in the differential form) for the marginal distribution. It is in this viewpoint that we say the limiting mean-field equations given in equation (A 5) satisfy the Chapman–Kolmogorov equations for the probability distribution *p*_*t*_. It is worth mentioning that the time derivative (d/d_*t*_)*p*_*t*_ gives us what is popularly known as the chemical master equation (CME) in the physical sciences literature. Note that our Chapman–Kolmogorov viewpoint is somewhat different from the notion of a generalized master equation (GME) [23,24] in that we are not attempting to describe the original stochastic system in §2 with equation (3.11), but rather constructing a Markov chain whose Chapman–Kolmogorov equations are given by the mean-field limit of the original stochastic process.

Viewing the limiting trajectory equations as satisfying Chapman–Kolmogorov equations also reveals that, if we have data only on individual infection times, the likelihood function ℓ_*I*_ in equation (3.2) is essentially a Markov likelihood function. Here, we described the Chapman–Kolmogorov viewpoint on the simplistic state space $\left\{S,S^{c}\right\}$. In general, we could construct a Markov process W(t)∈W :={S,I,R}×[0,∞) that keeps track of the immunological status along with the age of the individual. Accordingly, DSA can be shown to be tantamount to describing the transition kernel for *W*(*t*) in terms of the mean-field trajectory equations *x*_*t*_ and their densities *y*_*S*_, *y*_*I*_, *y*_*R*_. Since this viewpoint is only a side note and not the main aim of the paper, we leave the discussion for a future work. We do, however, refer interested readers to [25], where the authors use a stationary GME with memory terms and show that the effect of molecular memory is equivalent to the introduction of a feedback in the context of intracellular reaction processes. We also remark that in certain examples, e.g. [26], the non-Markovian formulation can be shown to be equivalent to a Markovian formulation in that the steady state of the non-Markovian process can be reduced to that of an equivalent Markov process.

### 3.5. Estimate of effective population size

In addition to giving a simple product-form likelihood function for *θ*, DSA also gives a ready estimate of the effective population size. Given *k*_*T*_, the number of cases observed by time *T*, the effective population size can be estimated by the discount estimator

3.12 $${\hat{n}}_{T}\;:=\frac{k_{T}}{1-z_{S}(T)}.$$

In similar vein, we can also estimate the final size of the epidemic as follows:

3.13 $${\hat{k}}_{\infty }=\frac{\tau k_{T}}{1-z_{S}(T)}.$$

Refer to [9,17] for further discussions on this.

## 4. Numerical results

In this section, we demonstrate how the DSA method can be used for inference of model parameters from infectious disease outbreak data using the likelihood functions described in §3. Typical outbreak data consist of population-level aggregated counts (such as the daily number of newly positive cases). Hence, we use this scenario as a benchmark for numerical validation. At the beginning, we will analyse synthetic data and make several simplifying assumptions, which we will gradually remove in favour of more realistic models when considering datasets from real epidemic outbreaks, such as the FMD epidemic in the UK and the COVID-19 pandemic in India.

### 4.1. Synthetic data

We begin by carrying out DSA analysis on synthetic data. We begin by keeping the premise deliberately simple: we assume that the family of the infectious period is known in that the functional form of the hazard function *γ* (or the PDF characterized by *γ*) is known, but the parameters are to be inferred along with the initial condition of the PDE (A 5) and a constant infection rate, *β*. To this end, we begin by assuming the infectious period is a Gamma random variable. The rationale behind this choice is the flexibility of the Gamma distribution and its historical importance in the infectious disease epidemiology, as a natural generalization of the Exponential distribution [27–31]. The proposed inference scheme, of course, works for any other distribution, such as the log-logistic or Weibull (see §4.2). All the code to reproduce the results in this section is available online,^2^ and a brief description of the numerical scheme used to solve the PDE can be found in appendix B.

#### 4.1.1. Description of data

The Sellke construction is an excellent means to generate exact simulations of an epidemic. We simulate an outbreak on a population of *N* = 10 000 individuals. Epidemics are run until no infected individuals are present in the population. Datasets consist of the series of infection and recovery times taken from the simulation, without noise nor delays.

We consider three different scenarios, characterized by different availability of data: we either work with only recovery times, with only infection times, or with both. We generate 1000 datasets from the same initial conditions, to characterize the distribution of the estimates. Estimates are found by means of a mix of global and local optimization routines.

**Figure 2.**
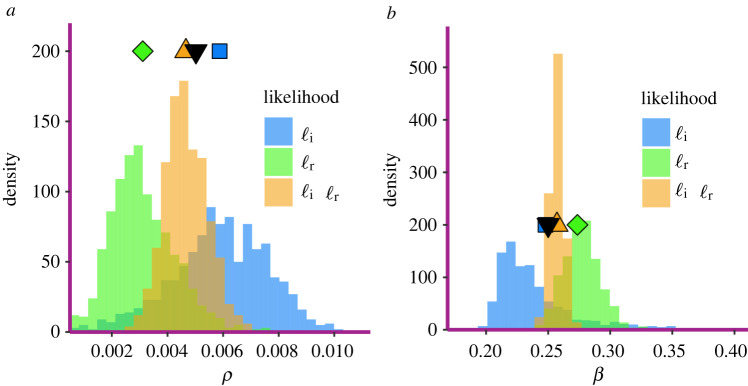
Inferred parameters (*a*) *ρ* and (*b*) *β*. Each figure shows histograms for different scenarios of data availability, as denoted in the legend. In each figure part, the true parameter is represented by the downward triangle; the square is the average value inferred when considering only infectious times, the diamond when considering only recovery times and the upward triangle when considering both.

**Figure 3.**
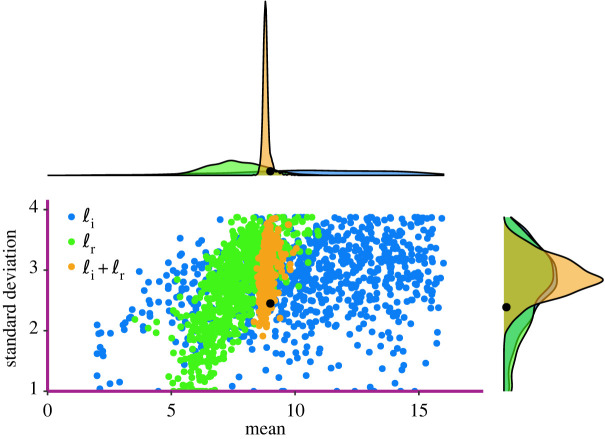
Inferred infectious period distribution mean and standard deviation. Black dots represent the true values.

The objective is to infer the initial proportion of infected individuals *ρ* = 50/9950, the per-contact infection rate *β* = 0.25, and the parameters of the distribution of infectious period, which is a Gamma distribution with mean *μ* = 9 and variance *σ*^2^ = 6. Results are shown in figures 2 and 3.

We find that inference based on only infection times using the likelihood function ℓ_*I*_(*θ*) in (3.2) results in wider distributions for all inferred parameters, suggesting greater uncertainty, than inference based on both. This is expected because the likelihood function ℓ(*θ*) in (3.7) is more informative than the likelihood function ℓ_*I*_(*θ*) in (3.2). In general, the true parameters are always near the mode of the distributions of the inferred parameters. It is worth noting that when the infection rate *β* is overestimated, the initial proportion of infected individuals *ρ* is underestimated, and *vice versa*. This suggests a potential statistical unidentifiability of the parameters. Outbreaks starting with a higher number of infected individuals but smaller transmission rate may be hard to distinguish from those that start with a smaller number of infected individuals but with higher transmission rate.

The mean and the standard deviation of the distribution of the infectious period are reported in figure 3. We observe that inference based only on infection times, in general, accurately captures the mean of the distribution of the infectious period but tends to overestimate the variance. The overall quality of inference improves significantly when recovery times are also available.

### 4.2. Foot-and-mouth disease

Let us now turn to real datasets. We consider the 2001 FMD outbreak in the UK. The outbreak began at the end of February 2001 and ended in September 2001, affecting more than 2000 farms. Policy makers' efforts to control the epidemic resulted in the culling of millions of herds and flocks [32]. Because of the specific interventions taken to control the outbreak, we interpret the infectious period in the DSA model as the time from when the disease hit a farm to the elimination of infected herds, i.e. the time to removal. Since this quantity is unlikely to be exponentially distributed, we fit a more flexible Gamma distribution to it. For the contact interval distribution characterized by the hazard function *β*, we assume a Weibull distribution, which is in line with other methods present in the literature [33]. We note that both these choices may be viewed as generalizing the usual Markovian model based on two Exponential distributions.

**Figure 4.**
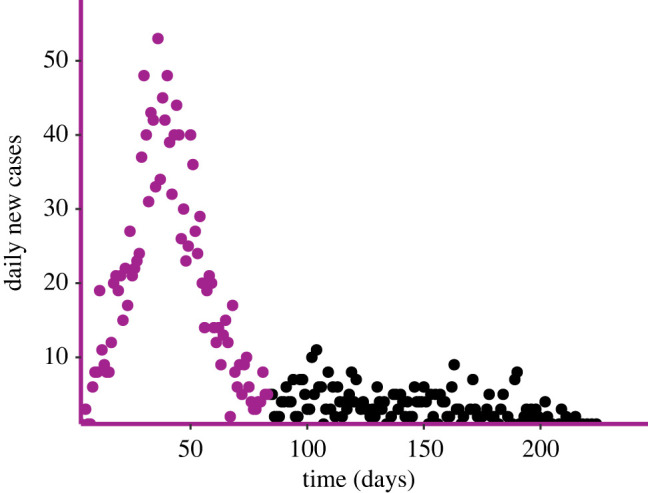
Visualization of the FMD outbreak. New daily cases since the first day of data (February 2001), to last day where a new case was confirmed (September 2001). The data points in black are excluded from the analysis.

The dataset^3^ consists of daily incidence of infected premises by time of report, {*t*_*i*_, *I*_*i*_}, with no information on removal times (figure 4). For each day *t*_*i*_, we distribute the number of new cases *I*_*i*_ uniformly in the interval (*t*_*i*−1_, *t*_*i*_). Furthermore, we consider only the first 80 days of data, to exclude the noisy tail and potentially confounding effects of strict measures. These simplifying assumptions allow us to maximize the likelihood ℓ_*I*_(*θ*) in (3.2). Since the original data points are too noisy, we consider the 7-day moving average of the counts, starting from day 6. This results in a smoother dataset that is less noisy, although a bit delayed with respect to the true one.

**Figure 5.**
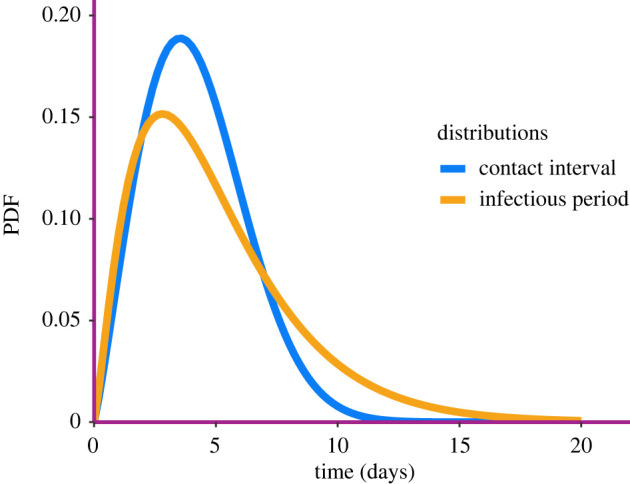
The best-fitting PDF of the contact interval and the infectious period inferred from the FMD data.

Maximum-likelihood estimates are obtained by means of a mix of global and local optimization routines. The distributions of inferred contact interval and infectious period are shown in figure 5. The shapes of the inferred distributions are in line with findings from other studies of same outbreak [34]. Our model with Weibull contact interval distribution and Gamma infectious period does not consider the incubation period explicitly. Once both infectious period and contact interval distributions are known, we can find *R*_0_ using the formula $R_{0}=\int_{0}^{\infty }S_{\gamma }(t)\beta (t)dt$ [35], where *S*_*γ*_, we recall, is the survival function of the infectious period distribution. This gives a point-estimate of *R*_0_ = 2.55. Finally, the effective population size inferred was ${\hat{n}}_{T}=2284$.

We compute confidence intervals using a bootstrap method, which we describe now. We first solve the limiting PDE (A 5) with the MLE estimates. From the solution, we compute the distribution of infection times PDF (3.1). This distribution is used to generate *n* = 500 synthetic datasets with as many datapoints as the original one, consisting of simulated dates of infections, on which we repeat the inference. Each new set of inferred parameters is then used to produce both the estimate for *R*_0_ (shown in figure 11) and the (*t*, *I*(*t*)) incidence curve that we can compare against the true data.

**Figure 6.**
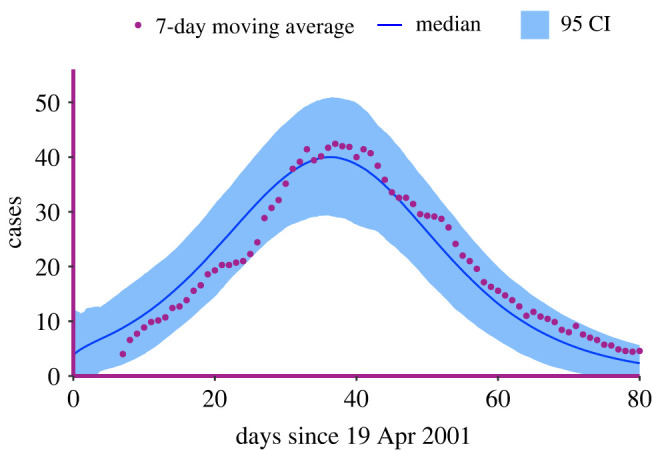
Variance-adjusted confidence intervals for the FMD dataset.

Finally, when computing confidence intervals, we compensate for other sources of noise that cannot be explicitly accounted for in our the model but are present in real-world data, such as testing limits, day-of-the-week effects and various sources of delays. This variance-adjustment is done by inflating the confidence intervals by a factor determined by taking the square root of the variance between the data points and the 7-day moving average. Results are shown in figure 6. As can be verified, the trajectories do capture the epidemic trend quite well in that all the data points lie within the variance-adjusted 95% confidence interval.

### 4.3. Comparison with Markovian compartmental models

**Figure 7.**
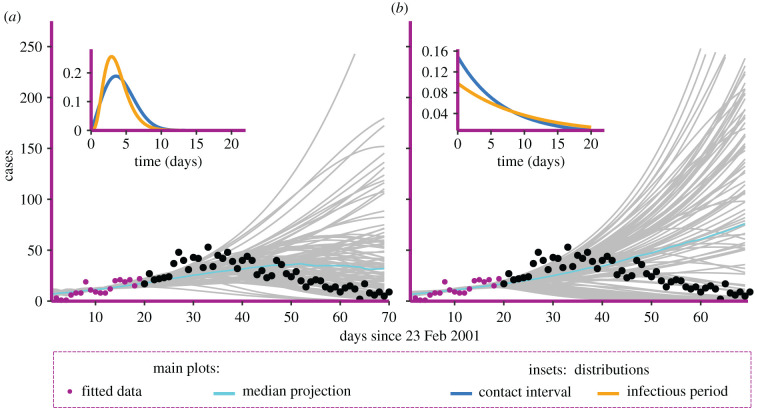
Comparison between (*a*) Weibull–Gamma model and the (*b*) Exponential–Exponential model. Only the first 20 days of reported cases are considered for the fitting, and the solutions (grey curves) are compared with the observed incidence (black dots) in the following 50 days of data. In the insets, the distributions are inferred from the MLE estimates. These distributions are Weibull(shape = 3.163, scale = 4.153), Gamma(shape = 4.675, scale = 0.8). For the standard exponential models, we have an average contact interval period of 6.7 days and average time to removal of 10.2 days.

We show the difference between a flexible non-Markovian model and the standard Markovian compartmental SIR model (with Exponential contact intervals and Exponential infectious periods) by comparing their performance for the FMD epidemic. Both models tend to perform better when the data on the full course of the epidemic are considered (not shown). Here, we present an analysis based on the more realistic situation where only early data are available. We are interested both in inference of the infectious period and the contact interval distributions, and in forward predictions. For the purpose of prediction, we include in our model only observations from the first 20 days, corresponding to roughly 300 cases, with a peak daily case count of 22 infected premises. The first three days are excluded from the inference because there were no cases reported on day 2 and day 3. It is worth remembering that the animal culling policy was introduced on 15 March 2001, which is after the last data point considered, although a national movement ban was already in place for the whole period. In both scenarios, we consider the curves obtained from the MLE estimates projected to day 70 and compared with real case count and cumulative infections. Results are shown in figures 7, and figure 13 in appendix C.

The DSA model is better able to capture the dynamics of the real epidemic, and gives substantially more reliable predictions, even when only a few observations are taken into account. Another important aspect is the interpretability of the results. The DSA model allows us to get a realistic idea of how both the contact interval and the infectious period distributions appear, whereas standard Exponential–Exponential models do not provide much insight, beyond looking at the rate of the underlying Exponential distributions. Another important result that the DSA model is able to achieve is the estimation of the effective population size. This can be interpreted as the number of farms that were potentially involved in the dynamics, and it is therefore an upper limit on the total number of farms that might become infected. For this model, the median estimated population size was ${\hat{n}}_{T}=5739$ (90% confidence interval [1740 − 32 224]; see also figure 14), in line with results from the literature [34].

### 4.4. Third wave of COVID-19 in India

The analysis of FMD outbreak data makes use of only infection times. As the synthetic data analysis suggests that inference based only on infection times tends to be poorer compared to when both infection times as well as recovery times are available, we now analyse an epidemic where both times are available.

**Figure 8.**
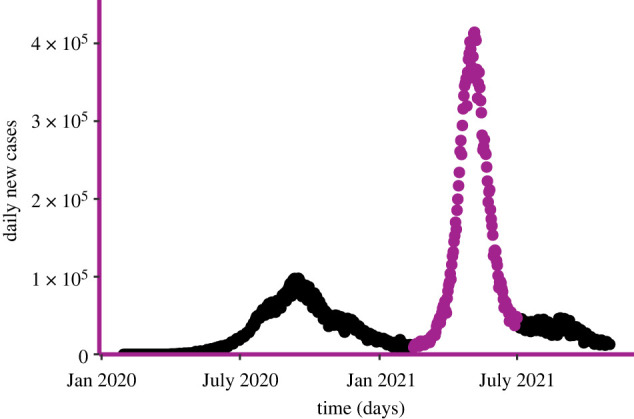
Indian wave of COVID-19 cases. The Delta-wave to which we fit the model is highlighted in purple, and spans from 15 February 2021 to 31 June 2021.

In a global effort to document and control the ongoing COVID-19 pandemic, many governments provided freely available population-level datasets that we can use as case studies for inference when both infection and recovery times are known. Various countries adopted strong non-pharmaceutical measures that drastically changed the local dynamics of the epidemic, resulting in several distinct epidemic waves. At the same time, new SARS-CoV-2 variants emerged with markedly different epidemiological characteristics. To curtail the impact of such exogenous factors, we consider only the third wave in India.^4^ Data consist of daily incidence and prevalence of cases, recoveries and deaths, meaning that we have data to inform both likelihoods in (3.2) and (3.6). The observed period spans from 15 February 2021 to 31 June 2021 inclusive (figure 8). For this dataset, we assume both the contact interval and the infectious period to be Gamma distributed.

Similar to our approach on the FMD data, daily cases are distributed uniformly across the day. Because the DSA method requires only a random sample of infection and recovery times, we work with a dataset generated by taking a random sample (without replacement) of size 3000. We do not consider exogenous factors such as under-reporting of cases as they are beyond the scope of this paper. It is worth noting, however, that these exogenous factors surely have an impact on the results and can be accounted for by a more refined model.

**Figure 9.**
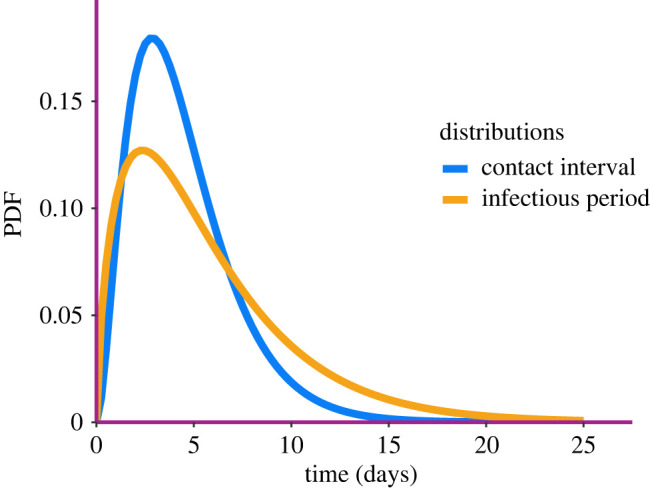
The best-fitting contact interval and infectious period distributions inferred from Indian Delta wave data. The distributions are, respectively, Gamma(4.5, 10), and Gamma(5.5, 20).

The best-fitting inferred contact interval and infectious period distributions are shown in figure 9. There are roughly in line with estimates of viral load and recovery distributions, respectively, from the literature [36]. The point estimate for the reproduction rate is *R*_0_ = 1.69. Although *R*_0_ of the SARS-CoV-2 Delta variant is estimated to be in the range 3–8 [37], it is more realistic to compare our estimate with *R*_*t*_ calculated from observed cases in that period, as our model uses only that source of information. The recovery distribution has a mean of 5.6 days and a variance of 26 days, so it is rather wide and right-skewed. The contact interval distribution is more peaked, with a slightly lower mean (around 4.5 days) and a variance of roughly 10. It is important to notice that infection times represent the collection of specimens from infected individuals, and recovery times follow country-specific healthcare system protocols, so they do not necessarily coincide with the true infectious distributions. Furthermore, the infectious period starts immediately after the incubation time has passed, while time to recovery is usually calculated from the onset of symptoms. Finally, the estimated effective population size is 31 million people. This is likely an underestimate because of the underreporting of cases in India during the Delta wave.

**Figure 10.**
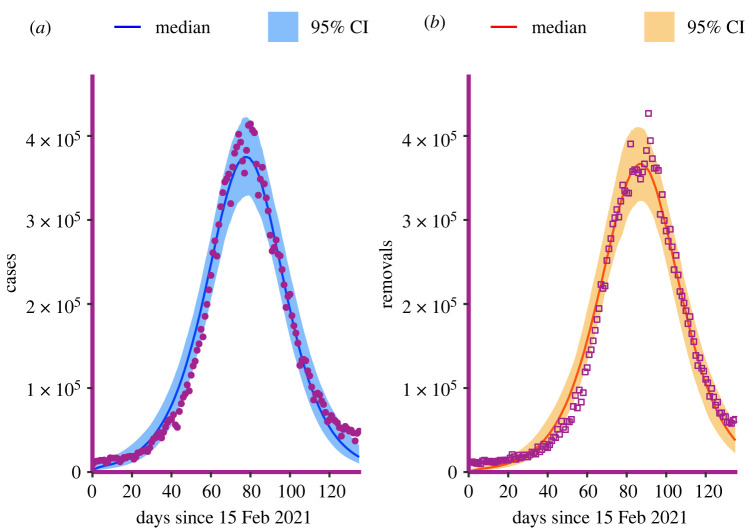
Confidence intervals for Indian wave. (*a*) Daily number of new cases, (*b*) daily number of recoveries or deaths (referred to as removals).

**Figure 11.**
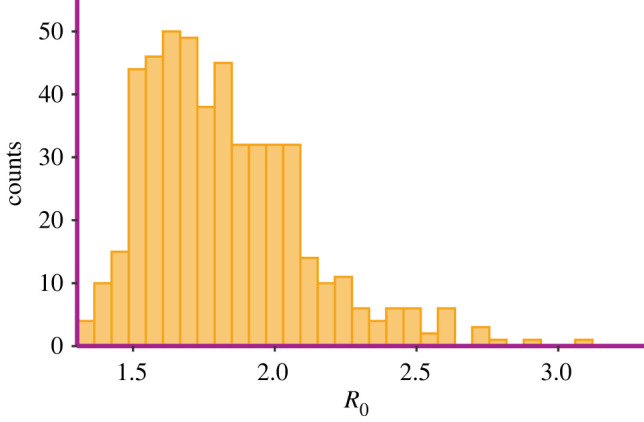
FMD *R*_0_ estimates from the bootstrap method.

**Figure 12.**
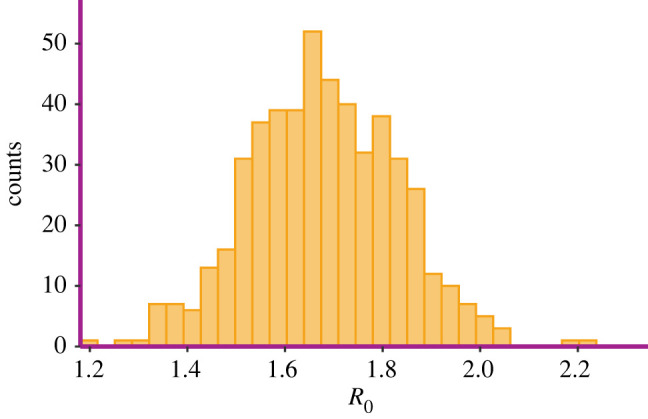
COVID-19 *R*_0_ estimates from the bootstrap method.

**Figure 13.**
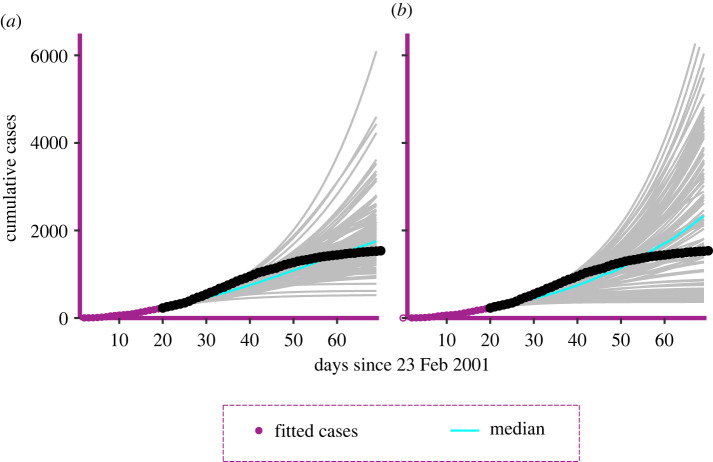
Comparison of cumulative incidence between (*a*) Weibull–Gamma model and standard stochastic (*b*) Exponential–Exponential model.

**Figure 14.**
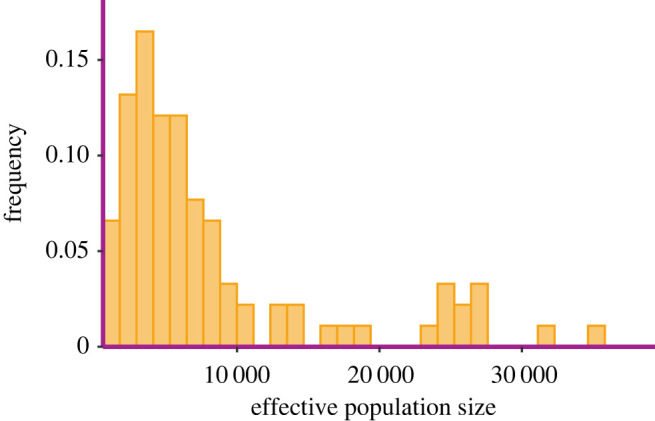
Frequency histogram of effective population size estimated from the first 20 days of data for the FMD.

Confidence intervals are computed in a similar way to the FMD analysis, with two major differences: the 7-day moving averages result in a curve that is too delayed with respect to the actual one because of exponential growth/decline. Although this effect may be accounted for by considering exponential moving averages, we preferred not to modify the data that way. For a similar reason, computing the variance-adjusted confidence intervals that take into account all the noise that cannot be explained by the model is not possible. Therefore, the confidence intervals, displayed in figure 10, underestimate the true variability of the underlying process, but seem to be generally in good agreement with the data. Interestingly, repeating the inference on different subsets of the original dataset does not produce significantly different estimates for the two distributions of interest. This suggests that the method is robust, not only because we have many data points to inform the likelihood, but also because we consider both the infection times and the recovery/death times. The distribution of the estimates of the reproduction number is shown in appendix C (figure 12).

## 5. Discussion

In this paper, we presented a method called DSA to both model and infer parameters of non-Markovian epidemic models. A crucial advantage of DSA is that it makes the entire toolkit of survival analysis available for making inference on dynamical systems. Therefore, DSA handles censored, truncated data in a straightforward and principled way. For instance, see [20] for an application of the DSA method adapted to a simple Markovian susceptible–exposed–infected–recovered (SEIR) model, where a snapshot of COVID-19 positivity data gathered through mass testing is used to analyse transmission in an Ohio prison. The analysis helped uncover the grave COVID-19 situation in correctional facilities in Ohio. Also, see [18] where we used the DSA approach coupled with approximate Bayesian computation (ABC) method to quantify the population-level effect of the mass vaccination campaign against COVID-19 in Israel. The analysis further helped quantify the indirect effect of vaccination on the unvaccinated young population in Israel. In [19], the DSA method was used to analyse the individual-level epidemic data from the Ebola pandemic in the Democratic Republic of Congo, suggesting success of the ring vaccination and contact tracing efforts evident from much lower estimates of the effective population size than previous analyses.

In this paper, we adopted the law of mass-action to model the interactions among the individuals for the sake of simplicity. Under the law of mass-action, an infected individual can potentially infect any susceptible individual in the population. This is in contrast with network-based models, where infected individuals can only infect their neighbours (connections defined by the graph adjacency matrix) [17,37–39]. However, inferring the underlying network structure is a non-trivial task and often infeasible. This is particularly true when the underlying network exhibits complex substructures [41]. Therefore, the mass-action models are still routinely used despite being unrealistic in many epidemics. Nevertheless, an immediate future direction for us would be to develop the DSA methodology for a non-Markovian network model.

The crux of the DSA methodology lies in the change in perspective about dynamical systems—one that views them as describing probability distributions of times of infection and recovery, as opposed to describing (scaled) counts. As such, the method is completely general and could be quickly adapted to the particular setting of any infectious disease. We hope the software package [42] will help translate the DSA methodology into a useful practical tool in modern infectious disease epidemiology.

## Data Availability

The FMD dataset was kindly provided by Professor Michael Tildesley of the University of Warwick. The COVID-19 dataset was downloaded from https://api.covid19india.org/documentation/csv/. A Python implementation of the methods is provided in [42] as a GitHub repository.

## Endnotes

1 We treat the infection time as a transfer time from the susceptible to the infected compartment. Similarly, the recovery time is seen as a transfer time from the infected to the recovered compartment.

2
https://github.com/Zkeggia/DSA_refactor

3 Data on daily incidence kindly provided by Prof. Michael Tildesley, University of Warwick.

4 Data available at https://api.covid19india.org/documentation/csv/

## Appendix A. Brief derivation of the mean-field limit

We provide an intuitive derivation of the PDE limit discussed in §2 for the scaled stochastic process *n*^−1^*X*_*t*_. The proof follows a standard line of argument via the tightness-uniqueness route for Banach space-valued Markov processes. Similar (and more elaborate) derivations can be found in [12–16]. For the sake of completeness, we furnish a short overview of the main arguments here.

*Notational conventions*: we denote the sets of natural numbers, non-negative integers, real numbers and non-negative real numbers by $N,N_{0},R$ and $R_{+}$, respectively. The set of Borel subsets of a set *E* will be denoted by B(E). For a set *E*, we use the notation *D*([0, ∞), *E*) (or *D*([0, *T*], *E*)) to denote the space of *E*-valued càdlàg functions defined on [0, ∞) (or [0, *T*], for some *T* > 0). The stochastic processes that we consider in this paper will be elements of *D*([0, ∞), *E*) or *D*([0, *T*], *E*)) for some state space *E* and some time horizon *T* > 0 unless otherwise specified. The set-function *δ*_*x*_ is the Dirac measure, i.e. for a set *A*, the function *δ*_*x*_(*A*) takes value 1 if *x* ∈ *A* and 0 otherwise. For a vector of point measures *ν* : = (*ν*_1_, *ν*_2_, …, *ν*_*k*_), for some positive integer *k*, and a measurable function *f*, we use the notation〈〈*μ*, *f*〉〉 to denote

$$\langle \langle \nu ,f\rangle \rangle \;:=\sum_{i=1}^{k}\langle \nu_{i},f\rangle .$$

The indicator (or characteristic) function of a set *A* is denoted by $1_{\left\{A\right\}}$, i.e. $1_{\left\{A\right\}}(x)=1$ if *x* ∈ *A* and 0 otherwise.

Following the above conventions, the processes $X_{t}^{S},X_{t}^{I}$ and $X_{t}^{R}$ are finite, point-measures on $R_{+}$ with atoms placed on the individual ages. Therefore, we have the following self-consistency relations $N_{S}(t)=\langle X_{t}^{S},1\rangle =X_{t}^{S}(R_{+})$, $N_{I}(t)=\langle X_{t}^{I},1\rangle =X_{t}^{I}(R_{+})$ and $N_{R}(t)=\langle X_{t}^{R},1\rangle =X_{t}^{R}(R_{+})$, where 1 is the identity function. The process *X*_*t*_ is a Markov process with paths in $D([0,T],M_{P}{\left(R_{+}\right)}^{3})$ where *T* > 0 is a finite-time horizon and $M_{P}(R_{+})$ is the space of finite, point measures on $R_{+}$.

### A.1. Trajectory equations

In order to write down the trajectory equations for the components of *X*_*t*_, we need to fix a partial order on the ages so as to make statements such as ‘age of the *i*th individual’ unambiguous. Let us fix the ‘greater than or equal to’ relation on $R_{+}$. Now, for *i* = 1, 2, 3, …, we define maps $\sigma_{i}\;:\;M_{P}(R_{+})\to R_{+}$, which gives us the age of the *i*-individual (i.e. the *i*th atom of a finite, point measure). Therefore, $\sigma_{i}(X_{t}^{I})$ is the age of the *i*th infected individual at time *t*. In order to describe the interactions, we shall assume the stochastic law of mass action. Now, assuming there are only susceptible and infected individuals initially, we can write down the trajectory equations for the measure-valued stochastic processes $X_{t}^{S},X_{t}^{I}$ and $X_{t}^{R}$ as follows:

A 1 $$\begin{array}{rl} X_{t}^{S} & =\sum_{k=1}^{N_{S}(0)}\delta_{t+\sigma_{k}(X_{0}^{S})}-\int_{0}^{t}\int_{N}\int_{0}^{\infty }\delta_{t-s+\sigma_{i}(X_{s-}^{S})}1_{\left\{i\leq N_{S}(s-)\right\}}\\ & \;\times 1_{\left\{\theta \leq \langle X_{s-}^{I},n^{-1}\beta (\sigma_{i}(X_{s-}^{S}),∙)\rangle \right\}}Q_{1}(ds,di,d\theta ),\\ & X_{t}^{I}=\sum_{k=1}^{N_{I}(0)}\delta_{t+\sigma_{k}(X_{0}^{I})}+\int_{0}^{t}\int_{N}\int_{0}^{\infty }\delta_{t-s}1_{\left\{i\leq N_{S}(s-)\right\}}\\ & \;\times 1_{\left\{\theta \leq \langle X_{s-}^{I},n^{-1}\beta (\sigma_{i}(X_{s-}^{S}),∙)\rangle \right\}}Q_{1}(ds,di,d\theta )\\ & \;-\int_{0}^{t}\int_{N}\int_{0}^{\infty }\delta_{t-s+\sigma_{k}(X_{s-}^{I})}1_{\left\{i\leq N_{I}(s-)\right\}}1_{\left\{\theta \leq \gamma (\sigma_{i}(X_{s-}^{I}))\right\}}\\ & \;\times Q_{2}(ds,di,d\theta )\\ \mathrm{and}\;\; & X_{t}^{R}=\int_{0}^{t}\int_{N}\int_{0}^{\infty }\delta_{t-s}1_{\left\{i\leq N_{I}(s-)\right\}}1_{\left\{\theta \leq \gamma (\sigma_{i}(X_{s-}^{I}))\right\}}Q_{2}(ds,di,d\theta ), \end{array}\}$$

where *Q*_1_ and *Q*_2_ are independent Poisson point measure (PPM) with intensity measures d*s* × d*i* × d*θ* with Lebesgue measures d*s*, d*θ* on $R_{+}$ and a counting measure d*i* on N. The PPM *Q*_1_ keeps track of infectious contacts, while the PPM *Q*_2_ book-keeps the natural recoveries of infected individuals. The intensity function *β* is scaled by a factor of *n*^−1^ following the stochastic law of mass action [1,2].

### A.2. Assumptions

It is sufficient to assume that the global jump rates (in terms of the instantaneous intensity functions *β* and *γ*) of the Markov process *X*_*t*_ are bounded above by a positive, finite quantity and that the initial population size does not explode in the sense that $E[n^{-1}(N_{S}(0)+N_{I}(0))]<\infty$ in order to ensure the trajectory equation (A 1) admits a unique path-wise solution $\left(X_{t}^{S},X_{t}^{I},X_{t}^{R}\right)$. This follows from arguments similar to [13], theorem 2.5 (see also [11,12,15]). To see this, note that trajectories satisfying (A 1) can be simulated by means of a straightforward adaptation of the Doob–Gillespie algorithm, which can be summarized as follows. (i) Given an initial condition satisfying the technical assumptions, compute the next event time (either an infection or a recovery) by drawing an exponential random variable with rate equal to the global jump rate (total hazard) $\left(\int \langle X_{t}^{I},n^{-1}\beta (u,∙)\rangle \;X_{t}^{S}(du)+\langle X_{t}^{I},\gamma \rangle \right)$. (ii) Determine the event type by drawing a categorical random variable with probabilities equal to the ratios of the hazards of the individual events and the total hazard. A pseudocode for simulating a similar age-structured birth–death transformation system is given in [11].

In addition to the assumption of the global jump rates (in terms of the instantaneous intensity functions *β* and *γ*) of the Markov process *X*_*t*_ being bounded above by a positive finite quantity, we also assume that the intensity functions *β* and *γ* are continuous. In order to study the FLLN approximation of the scaled process *n*^−1^*X*_*t*_, we further assume a finite second moment condition on the initial population size. That is, we assume $\underset{n}{\sup}E[n^{-2}{\left(N_{S}(0)+N_{I}(0)\right)}^{2}]<\infty$. Finally, we assume the initial age distribution does not explode.

Note that the assumptions about the initial size of the population are satisfied because *n* is chosen to be the size of the initial susceptible population and *m*/*n* → *ρ* ∈ (0, 1), as mentioned in §2. With the above technical assumptions, we are now ready to study the moments of the stochastic process *X*_*t*_ and associated martingale processes.

### A.3. Moments and martingale properties

Note that the components $X_{t}^{S},X_{t}^{I}$ and $X_{t}^{R}$ of *X*_*t*_ satisfy the stochastic integral equations described in (A 1). Then, for a sufficiently large class of test functions *f* : (*a*, *s*) → *f*_*s*_(*a*), the component measure-valued processes satisfy

A 2 $$\begin{array}{rl} \left\langle X_{t}^{S},f_{t}\right\rangle & =\sum_{k=1}^{N_{S}(0)}f_{t}(t+\sigma_{k}(X_{0}^{S}))-\int_{0}^{t}\int_{N}\int_{0}^{\infty }f_{t}(t-s+\sigma_{i}(X_{s-}^{S}))\\ & \;\times 1_{\left\{i\leq N_{S}(s-)\right\}}1_{\left\{\theta \leq \langle X_{s-}^{I},n^{-1}\beta (\sigma_{i}(X_{s-}^{S}),∙)\rangle \right\}}Q_{1}(ds,di,d\theta ),\\ \left\langle X_{t}^{I},f_{t}\right\rangle & =\sum_{k=1}^{N_{I}(0)}f_{t}(t+\sigma_{i}(X_{0}^{I}))+\int_{0}^{t}\int_{N}\int_{0}^{\infty }f_{t}(t-s)1_{\left\{i\leq N_{S}(s-)\right\}}\\ & \;\times 1_{\left\{\theta \leq \langle X_{s-}^{I},n^{-1}\beta (\sigma_{i}(X_{s-}^{S}),∙)\rangle \right\}}Q_{1}(ds,di,d\theta )\\ & \;-\int_{0}^{t}\int_{N}\int_{0}^{\infty }f_{t}(t-s+\sigma_{k}(X_{s-}^{I}))\\ & \;\times 1_{\left\{i\leq N_{I}(s-)\right\}}1_{\left\{\theta \leq \gamma (\sigma_{i}(X_{s-}^{I}))\right\}}Q_{2}(ds,di,d\theta )\\ \mathrm{and}\;\;\langle X_{t}^{R},f_{t}\rangle & =\int_{0}^{t}\int_{N}\int_{0}^{\infty }f_{t}(t-s)1_{\left\{i\leq N_{I}(s-)\right\}}1_{\left\{\theta \leq \gamma (\sigma_{i}(X_{s-}^{I}))\right\}}\\ & \;\times Q_{2}(ds,di,d\theta ), \end{array}\}$$

For different choices of the test function *f*, (A 2) can be used to study various moments of the component measure-valued processes $X_{t}^{S},X_{t}^{I}$ and $X_{t}^{R}$. Moreover, (A 2) allows us to study certain martingale processes associated with the stochastic process *X*_*t*_. For susceptible, infected and recovered compartments, define the stochastic processes

A 3 $$\begin{array}{rl} M_{t}^{S,f} & =\langle X_{t}^{S},f_{t}\rangle -\langle X_{0}^{S},f_{0}\rangle \\ & \;-\int_{0}^{t}\int_{0}^{\infty }\left(\frac{\partial }{\partial a}f_{s}(a)+\frac{\partial }{\partial s}\;f_{s}(a)-f_{s}(a)\langle X_{s}^{I},\beta (a,∙)\rangle \right)\\ & \;\times X_{s}^{S}(da)ds,\\ M_{t}^{I,f} & =\langle X_{t}^{I},f_{t}\rangle -\langle X_{0}^{I},f_{0}\rangle \\ & \;-\int_{0}^{t}\int_{0}^{\infty }\left(\frac{\partial }{\partial a}\;f_{s}(a)+\frac{\partial }{\partial s}\;f_{s}(a)+f_{s}(0)\langle X_{s}^{I},\beta (a,∙)\rangle \right)\\ & \;\times X_{s}^{S}(da)ds\\ & \;-\int_{0}^{t}\int_{0}^{\infty }\left(\frac{\partial }{\partial a}\;f_{s}(a)+\frac{\partial }{\partial s}\;f_{s}(a)-f_{s}(a)\gamma (a)\right)X_{s}^{I}(da)ds\\ \mathrm{and}\;M_{t}^{R,f} & =\langle X_{t}^{R},f_{t}\rangle -\langle X_{0}^{R},f_{0}\rangle \\ & \;-\int_{0}^{t}\int_{0}^{\infty }\left(\frac{\partial }{\partial a}\;f_{s}(a)+\frac{\partial }{\partial s}\;f_{s}(a)+f_{s}(0)\gamma (a)\right)X_{s}^{I}(da)ds. \end{array}\}$$

Using the compensated PPM of the original PPM *Q*_1_ and *Q*_2_, we can show that the stochastic processes $M_{t}^{S,f}$, $M_{t}^{I,f}$ and $M_{t}^{R,f}$ are, respectively, zero mean, square integrable, càdlàg martingale processes with predictable quadratic variations of the order *n*^−1^. Here, we have used the fact that

$$f_{t}(a+t-s)=f_{s}(a)+\int_{s}^{t}\left(\frac{\partial }{\partial u}f_{u}(a+u-s)+\frac{\partial }{\partial a}\;f_{u}(a+u-s)\right)du.$$

The trajectory equation for the scaled process *n*^−1^ *X*_*t*_ can be written in a straightforward fashion by dividing both sides of (A 1). We can then write down moment equations like (A 2) for the scaled process *n*^−1^ *X*_*t*_ and also define the corresponding scaled martingale processes. Since the global jump rates are assumed to be bounded above by a positive finite quantity, the predictable quadratic variation processes vanish in the limit of *n* → ∞. Therefore, in the limit of *n* → ∞, we expect the scaled martingale processes to vanish, which, in turn, implies that the scaled process *n*^−1^ *X*_*t*_ converges to a deterministic, continuous function $x_{t}\;:=(x_{t}^{S},x_{t}^{I},x_{t}^{R})$. However, such a convergence can only be guaranteed along a subsequence. Moreover, we need to ensure the sequence of the scaled processes *n*^−1^ *X*_*t*_ is tight.

### A.4. Tightness of the scaled process and uniqueness of limit points

The two main instruments here are the Roelly criterion [43] and the Aldous–Rebolledo criterion [44]. As done in [15] or [13, Proposition 3.1], we can establish the required tightness by verifying the Roelly criterion in the vague topology and the Aldous–Rebolledo criterion for the sequence of the scaled stochastic processes *n*^−1^ *X*_*t*_. The limit points $x_{t}\;:=(x_{t}^{S},x_{t}^{I},x_{t}^{R})$ of the scaled process *n*^−1^ *X*_*t*_ can be identified by virtue of the martingale representation in (A 3). Indeed, the functions $x_{t}^{S},x_{t}^{I}$ and $x_{t}^{R}$ satisfy

A 4 $$\begin{array}{rl} & \left\langle x_{t}^{S},f_{t}\rangle =\langle x_{0}^{S},f_{0}\right\rangle \\ & \;+\int_{0}^{t}\int_{0}^{\infty }\left(\frac{\partial }{\partial a}\;f_{s}(a)+\frac{\partial }{\partial s}\;f_{s}(a)-f_{s}(a)\langle x_{s}^{I},\beta (a,∙)\rangle \right)x_{s}^{S}(da)ds,\\ & \left\langle x_{t}^{I},f_{t}\rangle =\langle x_{0}^{I},f_{0}\right\rangle \\ & \;+\int_{0}^{t}\int_{0}^{\infty }\left(\frac{\partial }{\partial a}\;f_{s}(a)+\frac{\partial }{\partial s}\;f_{s}(a)+f_{s}(0)\langle x_{s}^{I},\beta (a,∙)\rangle \right)x_{s}^{S}(da)ds\\ & \;+\int_{0}^{t}\int_{0}^{\infty }\left(\frac{\partial }{\partial a}\;f_{s}(a)+\frac{\partial }{\partial s}\;f_{s}(a)-f_{s}(a)\gamma (a)\right)x_{s}^{I}(da)ds\\ \mathrm{and}\; & \left\langle x_{t}^{R},f_{t}\rangle =\langle x_{0}^{R},f_{0}\right\rangle \\ & \;+\int_{0}^{t}\int_{0}^{\infty }\left(\frac{\partial }{\partial a}\;f_{s}(a)+\frac{\partial }{\partial s}\;f_{s}(a)+f_{s}(0)\gamma (a)\right)x_{s}^{I}(da)ds, \end{array}\}$$

for a sufficiently large class of test functions *f* : (*a*, *s*) → *f*_*s*_(*a*). Given that the initial measures $x_{0}^{S}$, $x_{0}^{I}$ and $x_{0}^{R}$ admit densities with respect to the Lebesgue measure, it can be shown that the functions $x_{t}^{S},x_{t}^{I}$ and $x_{t}^{R}$ admit densities with respect to the Lebesgue measure throughout a finite-time interval [0, *T*] for some *T* > 0. Denoting the densities of the functions $x_{t}^{S}$, $x_{t}^{I}$ and $x_{t}^{R}$ by $y_{S}(t,∙)$, $y_{I}(t,∙)$ and $y_{R}(t,∙)$ respectively, we can describe the densities in terms of the following system of PDE (e.g. [12,13]):

A 5 $$\begin{array}{rl} & (\partial_{t}+\partial_{s})\;y_{S}(t,s)=-y_{S}(t,s)\int_{0}^{\infty }\beta (s,u)y_{I}(t,u)du,\\ & \left(\partial_{t}+\partial_{s})\;y_{I}(t,s)=-\gamma (s)y_{I}(t,s\right)\\ \mathrm{and}\; & (\partial_{t}+\partial_{s})\;y_{R}(t,s)=0, \end{array}\}$$

with boundary conditions

A 6 $$\begin{array}{rl} & y_{S}(t,0)=0,\\ & y_{I}(t,0)=\int_{0}^{\infty }y_{S}(t,s)\int_{0}^{\infty }\beta (s,u)y_{I}(t,u)duds\\ \mathrm{and}\; & y_{R}(t,0)=\int_{0}^{\infty }\gamma (s)y_{I}(t,s), \end{array}\}$$

and initial conditions $y_{S}(0,∙)\;:\;R_{+}\to R_{+}$, $y_{I}(0,∙)\;:\;R_{+}\to R_{+}$ such that

A 7 $$\int_{0}^{\infty }y_{S}(0,s)ds=1\;\mathrm{and}\;\int_{0}^{\infty }y_{I}(0,s)ds=\rho .$$

We set *y*_*R*_(0, *s*) = 0 for all $s\in R_{+}$ in keeping with our assumption that initially there are no recovered individuals. One can interpret *y*_*S*_(*t*, *s*), *y*_*I*_(*t*, *s*) and *y*_*R*_(*t*, *s*) as the densities at time *t* of susceptible, infected and recovered individuals at age *s*. The limiting system of PDE in (A 5) is linear in *y*_*S*_, *y*_*I*_ and *y*_*R*_, but non-local.

Now, since we have assumed the global jump rates are bounded above by a finite-positive number, we can show that the solutions remain bounded on finite-time intervals. In order to prove the uniqueness of the solutions, we can show that the distance between two possible solutions must vanish by invoking Grönwall’s lemma and by virtue of the fact that the solutions remain bounded on finite-time intervals.

## Appendix B. Numerical scheme to solve the mean-field PDE

In this section we describe the numerical schemes used to solve the PDE equation (2.4). In numerical terms, equation (2.4) is, inside the domain, an advection equation with one spatial dimension, in which the characteristics move at velocity *U*(*x*, *t*) = 1, and with a forcing term given by the right-hand side term −*γ*(*s*, *t*)*y*(*s*, *t*). Such equations are well known and can be solved with an explicit Semi-Lagrangian scheme [45,46]. The potential source of numerical instability comes from the nonlinear non-local boundary condition equation (2.5). We have opted for a numerical scheme that combines the explicit Semi-Lagrangian approach inside the domain and an implicit method to treat the solution at the boundary. Note that at the boundary we need to compute a scalar quantity; therefore, implementing an implicit method does not have a notable impact on the run-time of the numerical scheme itself, while improving the stability of the solution.

We define a mesh with spacing Δ*X* = 1/*M* and Δ*T* = 1/*N*, and points *s*_*i*_ = *i*Δ*x* and *t*^*n*^ = *n*Δ*T*, with 0 ≤ *i* ≤ *M* and 0 ≤ *n* ≤ *N*. The discretized set of equations is then:

B 1 $$\frac{y(t^{n+1},s_{i})-y(t^{n},s_{i}-(\Delta X/\Delta T))}{\Delta T}=-\gamma \left(s_{i}-\frac{\Delta X}{2\Delta T}\right)y\left(t^{n},s_{i}-\frac{\Delta X}{\Delta T}\right)$$

and

B 2 $$\frac{x_{S}(t^{n+1})-x_{S}(t^{n})}{\Delta T}=-x(t^{n+1})\sum_{k=1}^{M}\beta y(t^{n+1},s_{k})\Delta x$$

and, for the boundary condition

B 3 $$y(t^{n+1},0)=x(t^{n+1})\sum_{k=1}^{M}\beta y(t^{n+1},k)\Delta x.$$

For simplicity, we use Δ*X* = Δ*T*, so that *s*_*i*_ − (Δ*X*/Δ*T*) = *s*_*i*−1_. In algorithm B.1, we outline our implementation of the code. This returns *z*_*s*_(*t*) and $z_{I}(t)=\int_{0}^{\infty }y_{I}(t,s)\;ds$. It is straightforward to modify it to return *y*_*I*_(*t*, *s*).

**Algorithm B.1** Pseudo code to solve the PDE

**Require:** γ(s),β,ρ,f (s), number_of_points, $T_{f}$

 1: time_mesh=[$n*T_{f}/$number_of_points for n in range(0,number_of_points)]

 2: space_mesh=time_mesh   ▷ declare space and time meshes

 3: dx=1/number_of_points       ▷ space step Δx

 4: dt=dx

 5: y=zeros[time_mesh]    ▷ allocate memory for I(t)

 6: Y=zeros[space_mesh]   ▷ Allocate memory to hold y[t][s] at every time step

 7: $x_{S}$=zeros[time_mesh]   ▷ allocate memory for S(t)

 8: x_s[0]=1    ▷ initial fraction of susceptible people

 9:

10: **for** s in space_mesh **do**

11:  Y=ρ f[s]       ▷ initial condition on y[0][s]

12:

13: **end for**

14:

15: A=zeros(space_mesh,space_mesh)

16: **for** s in space_mesh **do**

17:  $A[s][s-1]=1/(1+dx*\gamma [s-\frac{1}{2}])$  ▷ first order approximation of the PDE operator

18: **end for**

19: **for** t in time_mesh **do**

20:  Y=A*Y           ▷ PDE propagation

21:  intY=sum(β[s]*Y*dx)     ▷ $\int_{0}^{\infty }\beta (s)y(t+1,s)\;ds$

22:  x[t+1]=x[t]/(1+dx*intY)      ▷ update x(t)

23:  y[t+1]=sum(Y)*dx       ▷ I(t)=∫y(t,s)ds

24:  y[0]=x[t+1] * intY  ▷ update Y at boundary with implicit scheme

25:

26: **end for**

27: **return** *y* and *x*

## Appendix C. Additional figures for FMD and COVID-19

We report the estimates for *R*_0_ from the bootstrap analysis of the FMD data (figure 11) and COVID-19 Delta wave in India (figure 12) (see §4). Results are based on 500 bootstrap samples obtained from simulating infection/recovery times with parameters given by the MLE. Additionally, we report the cumulative incidence distribution for the prediction of the FMD compared to that of a standard SIR model (figure 13). Finally, we show the histogram of the distribution of effective population size from bootstrap analysis in figure 14.
